# The Mechanism and Management of Adverse Cardiac Reactions Induced by Immune Checkpoint Inhibitors Therapy

**DOI:** 10.1155/2020/8367651

**Published:** 2020-12-16

**Authors:** Fangning Rong, Kangting Ji, Yingpei Weng, Yangpei Peng

**Affiliations:** The Second Affiliated Hospital of Wenzhou Medical University, Wenzhou, Zhejiang, China

## Abstract

Immune checkpoint inhibitors (ICIs) therapy has recently been introduced to all kinds of cancers. The adverse reactions associated with this therapy have attracted much attention. The heart-related adverse reactions are mainly the immune-related myocarditis and heart failure. Cases of adverse cardiac reactions caused by ICIs therapy have been clearly reported. However, the pathogenesis of the adverse cardiac reactions remains unclear. Therefore, this article briefly reviews the mechanism and management of adverse cardiac reactions caused by ICIs therapy.

## 1. Background

In the past decade, the treatment of advanced malignancies has been revolutionized by the development of immunotherapy. Immune checkpoint inhibitors (ICIs) have been successfully used in the treatment of various tumors [1, 2]. Recently, several monoclonal antibodies against cytotoxic T-lymphocyte-associated antigen 4 (CTLA-4) (e.g., ipilimumab), programmed cell death protein 1(PD-1) (e.g., nivolumab and pembrolizumab), and programmed cell death protein ligand 1(PD-L1) (e.g., atezolizumab and durvalumab) have been introduced to treat many different types of cancers [3–5].

Both CTLA-4 and PD-1 are immunoglobulin superfamily (IgSF) proteins structurally homologous to CD28, but they may play a role in different stages of T-cell response [6, 7]. CTLA-4 is only expressed on T cells and has two ligands, B7.1 and B7.2. CTLA-4 mainly acts on the primary T cells of secondary lymphoid organs [7, 8]. PD-1 is mainly expressed on activated T cells, B cells, NK cells, myeloid cells, antigen presenting cells (APC), and regulatory T cells. PD-1 activates T cells by interacting with two ligands currently known, PD-L1 and PD-L2. Expressed in many nonlymphoid tissues, PD-1 plays an important role in inhibiting effector T-cell activation in these tissues [7, 9–11].

The long-standing immune monitoring theory proposes that cells and tissues are constantly monitored by an ever-alert immune system, which is responsible for recognizing and eliminating the earliest cancer cells and thus preventing nascent tumors. The tumor progression may be achieved through some effective mechanisms that evade this immune system monitoring [12, 13]. One of the mechanisms is that tumor cells can dysregulate the expression of immune checkpoint proteins to escape the surveillance of antitumor T cells. PD-1 and CTLA-4 regulate immune responses at different levels through different mechanisms. Blocking the clinical activity of antibodies to one of these receptors means that antitumor immunity can be enhanced at multiple levels [11].

However, while inhibiting tumor progression, ICIs also inhibit important regulatory sites in the body's immune system, triggering a series of inflammatory reactions in normal tissues of multiple organs [14]. Such inflammatory reactions are collectively referred to as immune-related adverse events (irAEs). A total of 36,848 toxic reactions to ICIs were reported in a summary of FDA data (the FDA Adverse Events Reports System (FAERS)) for 2017-2018, with cardiovascular events accounting for 6.2% (including premature cardiac deaths). Among these adverse cardiac reactions, immune-related myocarditis is more common, while pericardial disease, endocarditis, and others are relatively rare [15]. Most cardiotoxic effects appear to be of an inflammatory nature [16]. Recently, those cardiotoxic effects have also been confirmed in animal studies.

## 2. Study on the Mechanism of Adverse Cardiac Reactions

Tivol et al. created CTLA-4 deficient mice in order to clarify the function of CTLA-4 in vivo. These mice rapidly developed lymphoproliferative diseases, accompanied by lymphocytic infiltration and tissue destruction of multiple organs, especially severe myocarditis and pancreatitis, and died at 3-4 weeks of age [17]. This suggests that CTLA-4 plays a key role in downregulating T-cell activation and maintaining immune homeostasis. From this, we may presume that when CTLA-4 is blocked, peripheral T cells are activated and can spontaneously proliferate, possibly mediating fatal tissue damage.

Grabie et al. used cytotoxic T cells to induce myocarditis. In that experiment, a CD8+ T cell-introduced myocarditis model was selected. This model transferred CD8+ OT-1 cytotoxic lymphocytes (CTLs) to CMy-mOva mice to explore how PD-L1 regulated CTLs-mediated tissue damage in the heart. They found that the genetic deletion of PD-L1 and PD-L2 and the use of PD-L1 inhibitors caused transient myocarditis to develop into a lethal disease in mice [18]. This suggests an important role of PD-1 signal in protecting myocardium from autoreactive T-lymphocyte damage.

A Japanese team studied the role of PD-L1 and PD-L2 blocking antibodies in the development of acute viral myocarditis in mice. They found that PD-L1 blocking antibodies could increase the expression of IFN-*γ*, FasL, CD40L, perforin, and viral genomes in myocardial tissue in mice [19]. This suggests that PD-L1 (but not PD-L2) plays a key role in suppressing infection. Factors such as IFN-*γ*, FasL, CD40L, and perforin play an important role in the inflammatory response.

Okazaki et al. found that mice lacking PD-L1 could develop autoimmune dilated cardiomyopathy (DCM) and produce high-titer autoantibodies against cardiac troponin-I (cTnI), and the administration of cTnI monoclonal antibody in wild-type mice could cause cardiac dilation and cardiac dysfunction. The monoclonal antibody against cTnI stained the surface of cardiomyocytes and increased the voltage-dependent L-type Ca2+ current of normal cardiomyocytes [20, 21]. They proved that the deletion of PD-1 gene in mice could cause cardiomyopathy due to autoantibodies against cTnI which could induce cardiac dysfunction and expansion by chronically stimulating the influx of Ca2+ in cardiomyocytes.

Ji et al. established an ICIs-induced myocarditis model with cynomolgus monkey. They injected intravenous intermedia or nivolumab 20 mg/kg plus ipilimumab 15 mg/kg weekly into Chinese crab-eating macaque monkeys and euthanized them on day 29. The following results were observed in cynomolgus monkeys: (1) multiple organ toxicity and monocyte infiltration of various severities in the heart, colon, kidney, liver, salivary glands, and endocrine organs; (2) increased proliferation of CD4+ and CD8+ T lymphocytes, as well as increased activation of T cells in blood, spleen, and lymph nodes; (3) gene expression analysis found that in monkey heart tissues administered in ipilimumab and nivolumab, the expression of multiple chemokine receptors increased, including CXCR3-CXCL9/CXCL10 and CCR5/CCL5 chemokine axis molecules; (4) transcriptome data indicated that in the treated monkeys, 38% of the related genes in the antigen presentation pathways were upregulated; (5) morphologically, heart disease in monkey models was similar to the ICI-related myocarditis reported in humans. In addition, transcriptomics analysis showed increased T-cell migration and activation, phagocytosis, and antigen presentation in the monkey model heart. The monocytes infiltrated in the myocardium were composed mainly of T cells, with a small number of macrophages and occasionally B cells. This was related to the minimum degree of myocardial degeneration and the increase of cardiac troponin-I and NT-pro-BNP [22]. Their results suggested that the adverse cardiac reactions related to ICIs were mainly caused by the systemic hyperproliferation of T cells and their subsequent infiltration into various tissues, and the increased expression of corresponding chemokines would promote the occurrence of such reactions.

Quagliariello et al. studied the cytotoxic and proinflammatory properties of ipilimumab and nivolumab. In this experiment, cocultures of human cardiomyocytes and lymphocytes were exposed to ipilimumab or nivolumab; cell viability and expression of leukotrienes, NLRP3, MyD88, and p65/NF-kB were performed. C57 mice were treated with ipilimumab (15 mg/kg). They analyzed changes in fractional shortening, ejection fraction, and radial and longitudinal strain before and after treatments through 2D-echocardiography. Meanwhile, the expressions of NLRP3, MyD88, p65/NF-kB, and 12 cytokines were analyzed in murine myocardium [23]. This indicates that ICIs may induce proinflammatory cytokine storms in heart tissue through the cytotoxic effects mediated by NLRP3/IL-1*β* and MyD88 pathways.

In summary, we believe that the possible mechanisms of heart damage related to ICIs mainly include the following: (1) polyclonal expansion of T cells and potential antigen-specific cellular immune responses: T cells systematically overproliferate and subsequently infiltrate into the heart, causing damage to cardiomyocytes; self-reactive T cell clones and infiltrates directly cause tissue damage. (2) Production of autoantibodies: the production of autoantibodies against cTnI may cause cardiomyopathy by chronically stimulating the influx of Ca2+ in cardiomyocytes. (3) Increased expression of related factors: increased expression of chemokine axis molecules, such as CXCR3-CXCL9/CXCL1 and CCR5/CCL5, may promote systemic proliferation and chemotaxis of T cells; the expression of cellular functional mediators, such as granzyme K, A, and B, Fas ligand, and perforin, may increase the activity of CD8+ T cells. (4) Upregulation of related gene expression: upregulation of genes involved in antigen delivery pathways may play a role. (5) Through special signaling pathways: inducing cytotoxicity through NLRP3/IL-1*β* and MyD88 pathways.

## 3. Management of Adverse Cardiac Reactions

In general, most irAEs are not obvious and are at a low-grade (grade 1-2). However, some patients still have severe and even life-threatening immune abnormal toxic reactions of grade 3-4. If not treated in time, these severe irAEs may cause treatment interruption and result in death.

For adverse cardiac reactions caused by ICIs therapy, there are currently no standard diagnostic criteria or consistent biomarker monitoring guidelines [24]. Biomarkers or clinical characteristics of adverse cardiac events are not significantly different from those of autoimmune heart disease, so the diagnosis is more depend on medical history. However, the increase in circulating biomarkers cTn I and/or cTn T and BNP or NT-pro-BNP is still considered to be the most useful noninvasive diagnostic test [25, 26]. Gowen et al. presented their study at ASCO in 2017, where they compared the autoantibody profile of patients before and after ipilimumab treatment. Comparing the cohort with severe irAEs with those without irAEs, they found that 129 IgG autoantibodies were significantly different in the pretreatment serum. Most of them, target nuclear antigens and mitochondrial antigens, are rich in metabolic pathways. However, this test needs more clinical validation [27].

Brahmer et al. convened a multidisciplinary team to provide recommendations for patients with irAEs from ICIs treatment, through the analysis of randomized controlled trials and cases released from 2000 to 2017 [28]. For grade 1 toxicity, close monitoring of adverse reactions should be performed while continuing ICIs treatment. For most grade 2 toxicity, treatment with ICIs can be suspended until symptoms return to grade 1 or below, and corticosteroids can be used. Grade 3 toxicity usually requires suspending ICIs therapy and starting the treatment of corticosteroids. High-dose corticosteroids (prednisone 1 to 2 mg/kg/d or methylprednisolone 1 to 2 mg/kg/d) were used at the beginning and reduced gradually within 4 to 6 weeks. If the symptoms do not improve within 24 hours or the patient is hemodynamically unstable (hypotension, malignant arrhythmia, sudden decrease in LVEF, etc.), methylprednisolone of 500–1000 mg/day should be intravenously injected immediately. Some refractory cases may require infliximab or other immunosuppressive therapy. Grade 4 toxicity recommends permanent discontinuation of ICIs treatment [28–30]. The management of cardiovascular irAEs of patients treated with ICIs is also proposed on this basis (see Table 1 for details).

When the adverse cardiac reactions are severe, the first option is to use steroids. Other immunomodulatory drugs (IMM) used in steroid-refractory cases include the anti-TNF-*α* antibody (infliximab), the antimetabolite (mycophenolate mofetil), and the calcineurin inhibitors (tacrolimus and cyclosporine prime) [31]. As mentioned above, the most important mechanisms of ICIs-related cardiac adverse reactions are polyclonal expansion of T cells and inflammation caused by the potential antigen-specific cellular immune responses. These IMM drugs can directly affect T cells or inhibit the effects of inflammatory factors through various pathways, thereby effectively alleviating adverse reactions such as immune myocarditis (see Table 2 for details).

## 4. Discussion

Recently, ICIs therapy has led to dramatic advances in cancer treatment. While inhibiting tumor progression, ICIs also trigger a series of inflammatory reactions in normal tissues, leading to various irAEs. Few studies, however, have examined the adverse cardiac events associated with ICIs. Therefore, our article mainly summarizes and explores the mechanism and management of adverse cardiac reactions caused by ICIs through previous animal experiments.

Our article has some limitations. Firstly, due to the lack of relevant experimental research, the discussion of specific mechanism in this article is not comprehensive enough. Secondly, in the absence of relevant clinical trials, it is not clear whether these mechanisms proved by animal experiments can be analogized to humans. Thirdly, this article also summarizes the management for heart-related adverse reactions, including biomarkers for diagnosis and treatment. But more clinical trials are needed to confirm our conclusions.

In short, further researches are needed on the mechanism and management of adverse cardiac events induced by ICIs therapy. For example, there is no recognized standard animal model that can be used for preclinical studies of adverse cardiac reactions associated with ICIs. Another example is whether ICIs therapy is suitable for cancer patients with a history of heart failure or myocarditis and whether it worsens the heart disease in these patients.

In addition, beyond what is discussed in this article, ICIs have much more parts worth studying. Except for CTLA-4 and PD-1 pathways, there are now a large number of costimulatory and coinhibitory pathways. These pathways fall into two major families: the Ig superfamily, which includes the B7-CD28, TIM, and CD226-TIGIT-CD96 families as well as LAG-3, and the TNF-TNF receptor superfamily [6]. These pathways are likely to become new research directions for ICIs. Of course, when developing new ICIs, perhaps more attention should be paid to reducing the adverse effects of ICIs therapy.

## 5. Summary

Immune checkpoint inhibitor therapy, as a major breakthrough in cancer treatment, has become a research hotspot in recent years. As the number of patients receiving this therapy continues to increase, reports of related adverse reactions are also increasing. Further research is needed for the safe and effective use of ICIs.

## Figures and Tables

**Table 1:** Management of cardiovascular irAEs in patients treated with ICIs [28].

Grading	Management method
G1, abnormal detection of cardiac biomarkers, including abnormal ECG G2, abnormal screening with mild symptoms G3, moderate abnormal test or mild activity symptoms G4, moderate to severe decompensation, requiring intravenous medication or intervention, life-threatening condition	Patients of all levels should consider the possibility of heart damage and conduct examinations and interventions, mainly considering the following points: (i) Suspend or terminate ICIs treatment above G1 level (ii) Use of high-dose corticosteroids (prednisone 1-2 mg/kg) (oral or intravenous depending on symptoms) (iii) Manage cardiac symptoms according to ACC/AHA guidelines and under the guidance of cardiology (iv) Patients with elevated troponin or abnormal conduction are immediately transferred to the coronary care unit In patients who do not respond immediately to high-dose corticosteroids, consider an early heart transplant rejection dose of corticosteroids (methylprednisolone 1g per day), plus mycophenolate, infliximab, or antithymus cytoglobulin

**Table 2:** Immunomodulatory drugs and mechanism of action [31].

Drug	Main mechanism of action
Steroid	By inhibiting interleukin transcription, reducing cytokine synthesis, inhibiting neutrophil apoptosis, and reducing macrophage function, it has multiple effects on T cells, B cells, and phagocytic cells
Infliximab	Antibody that inhibits the binding of inflammatory cytokine tumor necrosis factor alpha (TNF-a) to its receptor
Mycophenolate mofetil	Inhibition of inosine monophosphate dehydrogenase (IMPDH) and inosine monophosphate dehydrogenase
Tacrolimus and cyclosporine	Calcineurin inhibitors can limit the transcription of interleukin 2 (IL-2) and participate in T-cell proliferation

## Data Availability

No data were used to support this study.
